# Utilization of Porcine Livers through the Formation of Zn-Protoporphyrin Pigment Optimized by a Response Surface Methodology

**DOI:** 10.3390/foods12091903

**Published:** 2023-05-06

**Authors:** Mar Llauger, Jacint Arnau, Michela Albano-Gaglio, Sara Bover-Cid, Belén Martín, Ricard Bou

**Affiliations:** 1 Food Safety and Functionality Program, Institute of Agrifood Research and Technology (IRTA), Finca Camps i Armet s/n, 17121 Monells, Spain; mar.llauger@irta.cat (M.L.); sara.bovercid@irta.cat (S.B.-C.); belen.martin@irta.cat (B.M.); 2 Food Quality and Technology Program, Institute of Agrifood Research and Technology (IRTA), Finca Camps i Armet s/n, 17121 Monells, Spain; jacint.arnau@irta.cat (J.A.); michela.albano@irta.cat (M.A.-G.)

**Keywords:** porphyrin, offal, color, ingredient, heme

## Abstract

There is a growing demand for clean-label products. This study aimed to obtain a food-grade coloring ingredient for meat products based on the formation of Zn-protoporphyrin from porcine livers, thus contributing to the development of nitrite-free products. First, the effects of sodium disulfite and acetic, ascorbic, and lactic acids on the formation of Zn-protoporphyrin and the total microbial count were studied. The combination of ascorbic and acetic acids resulted in a higher Zn-protoporphyrin content than acetic acid alone, and microbial levels were maintained (ca. 3 log CFU/mL). Second, a response surface methodology was used to maximize Zn-protoporphyrin while maintaining microbiological food standards. To that end, the effects of pH (4.2–5.4), incubation time (3–30 h), and temperature (25–50 °C) were studied. The selected conditions for Zn-protoporphyrin formation involved anaerobic incubation at pH 4.8 and 45 °C for 24 h. The safety was validated through challenge testing for relevant pathogens (*Listeria monocytogenes*, *Salmonella* spp., and *Clostridium perfringens*). A significant reduction (>6 log units) was observed in the selected conditions for *L. monocytogenes* and *Salmonella*, whereas *C. perfringens* spores remained at the inoculated levels. The optimized procedure is proven to be microbiologically safe, and may improve the color of nitrite-free meat products.

## 1. Introduction

The color of meat products influences consumers’ purchasing decisions, as consumers judge the quality of fresh meat and meat products based on their color [1]. Nitrites and nitrates are widely used in the production of cured meat products because they contribute to microbiological safety and color development and stability through the formation of the pigment nitrosyl-myoglobin [2]. Despite their positive effects, their use as meat-curing agents is controversial because, under certain conditions, such as low pH and high temperatures, they can react with secondary amines to form N-nitrosamines with potential carcinogenic effects [3,4]. Moreover, in many countries, there is a growing demand for clean-label products amid concerns regarding the use of additives [5]. This trend has led the meat industry to seek out alternative strategies for obtaining nitrite-free and clean-label meat products while maintaining their characteristic red color.

In this context, it is relevant that the red pigment zinc protoporphyrin (ZnPP) is formed during the development of nitrite-free dry-cured ham, such as traditional Parma ham [6,7], which creates exciting opportunities for innovation. The ZnPP chromophore that is naturally formed during the manufacturing of dry-cured hams has been reported to be relatively stable under light, heat, and low oxygen concentrations [8]. Specific lactic acid bacteria have also been shown to form ZnPP during the manufacturing of fermented sausages [9]. However, the availability of meat products containing ZnPP formed during manufacturing is currently low and limited to dry-cured ham. However, these recent findings support the idea that meat products that either promote the formation of ZnPP or to which it is directly added during manufacturing can be an alternative to conventional nitrified meat products.

In dry-cured hams, the formation of ZnPP has mainly been attributed to the activity of the endogenous enzyme ferrochelatase (FECH) [10,11]. Under normal physiological conditions, this enzyme catalyzes the formation of heme by inserting ferrous iron into protoporphyrin IX (PPIX) [12]. However, under certain conditions, this enzyme can insert other divalent ions, such as zinc, which leads to the formation of ZnPP. Additionally, it is responsible for the enzymatic removal of iron ions from the heme moiety, suggesting that the formation of PPIX is a necessary first step in obtaining ZnPP from heme compounds [13,14]. The activity of FECH and the formation of ZnPP depend on various factors, including the muscle fiber type, type and origin of the muscle, and viscera [15,16]. De Maere et al. [15] reported the potential of horse meat and porcine liver to form ZnPP because of their high enzymatic activity. Indeed, the porcine liver was found to have the highest capacity to form ZnPP in various organs, including the heart, liver, and kidneys [17]. 

It is estimated that the liver and other co- and by-products of meat production constitute approximately 30% of the live weight of hogs, the disposal of which may have a significant impact from environmental and economic perspectives. These co-products are edible after appropriate cleaning, handling, and processing [18]. However, new consumption trends have decreased liver consumption and other offal products [19]. Therefore, new insights into porcine liver utilization would benefit the meat industry, reducing the global demand for protein sources and the environmental impact of managing this co-product. In this regard, using porcine livers to form ZnPP may represent an important advance for obtaining potential food coloring ingredients for the development of many nitrite-free meat products. However, liver and liver products are rich in nutrients, which makes them ideal for bacterial growth and limits their shelf life [20]. Because sulfites and various organic acids are well-known food preservatives, they may allow for the formation of ZnPP and control microbial growth. 

This study hypothesizes that obtaining food-grade coloring ingredients from porcine livers is possible by controlling pH and other factors. The response surface methodology (RSM) with a central composite design (CCD) is a practical approach to traditional optimization methodologies because it allows different parameters to be optimized simultaneously, minimizing the number of experiments [21]. Thus, this study aimed to optimize ZnPP formation to obtain coloring ingredients under conditions that control microbiological growth. Therefore, a preliminary investigation was conducted to investigate the effects of various organic acids and reducing agents on the formation of ZnPP and microbial growth. The effects of temperature, pH, and incubation time were evaluated using the selected conditions and RSM methodology to determine the optimal conditions for preparing ZnPP food-grade coloring ingredients. 

## 2. Materials and Methods

### 2.1. Reagents and Standards

Zinc (II) protoporphyrin IX (ZnPP), protoporphyrin IX (PPIX), adenosine 5′-triphosphate disodium salt hydrate (ATP), and ethylenediaminetetraacetic acid disodium salt dihydrate (EDTA) were purchased from Merck (Darmstadt, Germany). HPLC gradient-grade acetonitrile (ACN) and trifluoroacetic acid (TFA) were obtained from Merck (Darmstadt, Germany). Porcine hemin chloride (heme) was obtained from PanReac AppliChem (Barcelona, Spain). Food-grade ascorbic acid (E-300) was purchased from Collelldevall SL (Banyoles, Spain). All other reagents used in this study were of ACS grade. Distilled water was obtained using a Milli-Q system (Merck Millipore, Darmstadt, Germany). 

### 2.2. Materials

In the preliminary investigation with organic acids and reducing agents, 12 commercial porcine livers (4 randomly assigned to each triplicate) were collected on the same day from local slaughterhouses (Costa Brava SA, Girona, Spain). In the RSM study, sixteen commercial porcine livers were collected on the same day as slaughter from two local slaughterhouses (Costa Brava SA, Girona, Spain; Friselva Girona, Spain). Each replicate consisted of 4 randomly assigned porcine livers from each slaughterhouse (pooled porcine livers, n = 8). Similarly, 11 pooled porcine liver samples (66 livers in total) of the exact origin were collected for model validation and scale-up. Livers were immediately vacuum-packed and frozen at −20 °C until needed. They were partially thawed (≤0 °C), diced, and trimmed of veins and connective tissue. Subsequently, the diced liver was ground in a meat cutter bowl at ≤4 °C until a fine liver paste was obtained. The ground liver paste was vacuum-packed in metalized bags with polyethylene terephthalate/polyethylene packaging material (oxygen permeability of 1.5 mL/m^2^/24 h and low water vapor permeability of 1 g/m^2^/24 h) and stored at −20 °C until further use.

#### Standard Procedure for the Preparation of Porcine Liver Homogenates

Porcine liver homogenates were prepared using a method similar to the meat-based model described by Wakamatsu et al. [7], in order to examine their ZnPP formation capacity under different conditions. The homogenates were prepared under aseptic conditions. The final liver homogenate consisted of 20% (*w*/*w*) ground liver tissue. In brief, approximately 60 g of the ground porcine liver was homogenized with 4 volumes of distilled water using an UltraTurrax T25 model disperser (IKA, Werke GmbH & Co. KG, Staufen, Germany) at 9500 rpm for 1 min. The pH of the liver homogenates was adjusted to the desired final pH with an appropriate volume of NaOH 1 N and HCl 1 N. Aliquots (20 mL) were transferred to 50 mL Falcon tubes, capped with a sterile cellulosic cap to allow for air removal, and finally incubated at the desired temperatures and for the given times in the dark in an anaerobic jar containing an anaerobic gas generator (AnaeroGen™ 2.5 L, Oxoid, Basingstoke, UK).

### 2.3. Effect of the Addition of Disulfite and Organic Acids 

The effects of the addition of disulfite (DS, Na_2_S_2_O_5_; at 0, 750, and 1500 mg/L), lactic acid (LA; at 0, 1370, and 2715 mg/L), acetic acid (AcOH; at 0, 1250, 2500 mg/L), and ascorbic acid (AA; at 0, 250, 500, and 1000 mg/L) on the ZnPP-formation capacity of the liver homogenates were studied. DS and organic acids were dissolved in distilled water before homogenization to obtain the desired final concentrations. Similarly, the synergistic effect between AA and AcOH (1000 and 2500 mg/L, respectively) was also studied. Unless specified otherwise, the pH of the liver homogenates was adjusted with NaOH or HCl to 4.5. Subsequently, they were incubated under anaerobic conditions at 37 °C for 24 h. All experiments were conducted in triplicate.

### 2.4. Response Surface Methodology (RSM)

#### 2.4.1. Experimental Design

RSM with a central composite design (CCD) with three central points was employed to optimize the formation of ZnPP in porcine liver homogenates. Table 1 shows the three factors (pH, temperature, and incubation time) and five levels considered in the CCD. Based on the findings of preliminary experiments (previous section), all liver homogenates were prepared with AA and AcOH (final concentrations of 1000 and 2500 mg/L, respectively). The final pH of various aqueous solutions of AA and AcOH was adjusted to the desired pH before adding NaOH 1 N and HCl 1 N to prevent excessive dilution. Throughout the incubation, the temperature was recorded every 3 min using a data logger (PicoVACQ 1Tc, TMI-Orion, Reston, VA, USA) that was placed in a tube containing the porcine liver homogenate. Optimization of the response surface variables was aimed at maximizing the ZnPP-forming capacity and minimizing the counts of total viable microorganisms (TVC), which were evaluated as dependent variables (see Section 2.6 and Section 2.8). The resulting 17 runs (Table 2) were conducted in duplicate. 

#### 2.4.2. Validation of the Optimal ZnPP-Forming Conditions

Fourteen additional experiments were performed to validate the selected optimal conditions from the RSM. The first three confirmation experiments were performed using the same replicates as in the RSM experimental design under aseptic conditions, and the ZnPP content and total viable counts of aerobic mesophilic bacteria (TVC) were determined. The other 11 experiments used freshly prepared liver homogenates from 4–7 minced porcine livers pools. These latter experiments were used to examine the scale-up of the ZnPP formation process up to 4 and 8 L under pilot plant conditions (respectively, in 5- and 10 L-capacity reactors with a continuous flow of nitrogen and immersed in a water bath), and only the ZnPP content was assessed. After incubation, the porcine liver homogenate was concentrated by centrifugation at 5520× *g* for 20 min at 4 °C, and preliminary analysis showed that ZnPP remained in the insoluble fraction.

### 2.5. Porphyrin Determination

ZnPP was extracted under subdued light conditions, as described by Bou et al. [6], with minor modifications. Briefly, 1 g of liver homogenate was weighed, and 10 mL of ethyl acetate/acetic acid/dimethyl sulfoxide (10:2:1; *v*/*v*/*v*) was added. The mixture was shaken vigorously for 30 s using a vortex mixer. After extraction on ice for 20 min, the solution was centrifuged (1900× *g*, 15 min, 4 °C). The supernatant was filtered through filter paper and collected in an amber volumetric flask (typically 10 mL). Then, 200 microliters of the clear supernatant were transferred to 96-microwell plates and submitted to fluorescence analysis using a Thermo Fisher Scientific Varioskan Flash microplate reader (Waltham, MA, USA) with excitation at 416 nm and emission at 588 nm. Each sample was analyzed in triplicate and the mean value was kept as a single measurement.

The extracts obtained for ZnPP quantification were also used to determine the heme content by HPLC. The extract aliquots were filtered through a 0.45 µm PTFE syringe filter and injected (20 µL) into an Agilent 1100 series HPLC system (Waldbronn, Germany). Porphyrins were separated using a reverse-phase Luna C18 column (150 × 4.6 mm, 5 µm, 100 Å) from Phenomenex (Torrance, CA, USA) and detected using a UV/Vis detector set at 400 nm. Mobile phase A consisted of 0.05% TFA dissolved in Milli Q water, whereas mobile phase B consisted of 0.05% TFA dissolved in ACN. Porphyrins were eluted at a constant flow rate of 1 mL/min with a starting mobile phase of 90% A and 10% B, and then a linear gradient elution was run from 10% to 100% phase B for 15 min. Finally, the column was re-equilibrated with 90% A and 10% B for 5 min. Each sample was analyzed in triplicate and the mean value was kept as a single measurement. 

Porphyrin results were expressed on a dry-weight basis (DM), which was determined by drying at 103 ± 2 °C until a constant weight was reached, considering the recovery of 80% and 60% for ZnPP and heme, respectively.

### 2.6. Challenge Test with Pathogenic Bacteria

Two challenge tests were performed to assess microbial safety under the optimal conditions for ZnPP formation. First, a cocktail of three strains of *Listeria monocytogenes* (CTC1034 (IRTA’s collection, isolated from cured ham), Scott A (clinical isolate), and 12MOB045LM (EU Reference Laboratory for *L. monocytogenes*)) and three strains of *Salmonella enterica* (serovar London CTC1003 and Derby CTC1756 (IRTA’s collection, isolated from fermented sausages), and CCUG34136 (serovar Enteritidis)) were used. The cultures were prepared by independently growing each strain in Brain Hearth Infusion (Difco, Becton Dickinson and Co., Sparks, MD, USA) for 7 h and subculturing again for 18 h at 37 °C. The latter cultures were preserved at −80 °C in a cryoprotective solution (20% glycerol) and quantified before sample inoculation [22]. Second, a cocktail of three strains of *Clostridium perfringens* (CECT486 (isolated from boiled salt beef), CCUG (food poisoning outbreak), and CTC1768 (IRTA’s collection, isolated from porcine intestinal mucosa)) was used. The spores were produced independently in a fluid thioglycolate medium and Duncan-Strong sporulation medium [23], modified according to Labbe and Rey [24], to improve sporulation; they were then incubated for 22 h at 37 °C. The spores were concentrated by centrifugation (12,000× *g* for 10 min). After rinsing to eliminate vegetative cells and toxins, the spore precipitate was resuspended in 3 mL sterile distilled water and stored at 4 °C until use. Before sample inoculation, spores were subjected to thermal shock (75 °C for 20 min). 

The cocktails of *L. monocytogenes*, *Salmonella*, and *C. perfringens* (ca. 7 logs CFU/mL for each pathogen) were inoculated in three independent liver homogenates prepared under non-aseptic conditions and incubated under the selected optimal conditions for ZnPP formation (see Section 2.4.2). The studied pathogen population was quantified at four sampling points within the ZnPP formation process: (i) prior to inoculation, to verify the absence of the studied pathogens, (ii) after pathogen inoculation, (iii) after 24 h incubation, and (iv) after centrifugation (5520× *g*, for 20 min, 4 °C). For a better comparison, the pellet resulting from centrifugation was resuspended to the initial volume with distilled water. 

### 2.7. Microbiological Analysis

TVC was determined on Plate Count Agar (PCA, Oxoid, Unipath, Basingstoke, UK) incubated at 30 °C for 72 h. Results were expressed as log CFU/mL of the liver homogenate. *L. monocytogenes* was enumerated on CHROMagar™ Listeria (CHROMagar, Paris, France) incubated at 37 °C for 48 h. The enumeration of *Salmonella* was conducted on CHROMagar™ Salmonella (CHROMagar) incubated at 37 °C for 24 h. *C. perfringens* was enumerated on CHROMagarTM *C. perfringens* (CHROMagar), which was incubated at 37 °C for 24 h under anaerobic conditions (Anaerogen sachets, Oxoid). Microbial counts are expressed as log CFU/mL of the liver homogenate. The detection limit of all the assays was 1.30 log CFU/mL.

### 2.8. Zinc-Chelatase Activity

The zinc-chelatase activity of the FECH was measured as described by Parolari et al. [25]. Briefly, 2.5 mL of the homogenate was mixed with 2.5 mL of a double-concentrated ice-cold buffer (pH = 8) and 5 mL of an ice-cold standard buffer (pH = 8). The double-concentrated buffer extraction solution consisted of Tris buffer 100 mM, glycerol 40% (*w*/*v*), KCl 1.6% (*w*/*v*), and Triton X-100 2% (*w*/*v*), while the standard buffer contained a half concentration of all the reagents. After 30 min of gentle agitation at 4 °C, the sample was centrifuged (27,000× *g*, 10 min, 4 °C), and the supernatant was filtered through filter paper (grade 597). The Zn-chelatase activity assay used an aliquot (100 µL) of the filtrate. The filtrate was incubated at 37 °C for 45 min in the dark with 250 μL of 400 μM ZnSO_4_ in 360 mM Tris-HCl buffer (pH 8.0), 50 μL of 0.25 mM protoporphyrin IX in 360 mM Tris-HCl buffer (pH 7.0), and 200 μL of 25 mM ATP in NaCl 20% (*w*/*v*). Each extract was assayed against a blank sample obtained by adding 35 µL 50 mM EDTA to the reaction mixture. After incubation, the enzymatic reaction was stopped by adding 35 µL EDTA and placing the reaction tubes in an ice-cold bath for 1 min. Finally, the content of the reaction tubes was mixed with 635 µL of cold ethanol (96%), vortexed, and centrifuged (26,000× *g*, 10 min, 4 °C). The clear supernatant was analyzed by fluorescence using a microplate reader with excitation at 420 nm and emission at 590 nm. Each extract was assayed in triplicate. Zinc-chelatase activity was expressed as the amount of enzyme per gram of liver (DM basis) catalyzing the formation of 1 nmol ZnPP in 1 min. 

### 2.9. Statistical Analysis

Results are expressed as the mean ± standard error (SE). Statistical analyses were performed using the JMP^®^ 16.2.0 software (SAS Institute, Cary, NC, USA). 

We examined the differences among treatments involving the addition of DS and organic acids at the same incubation time and among incubation times within the same treatment by ANOVA using Tukey’s test. A probability of *p* < 0.05 was considered statistically significant. 

The optimization of ZnPP formation using RSM-CCD was performed by regression analysis using a polynomial model. This regression analysis was performed with the dimensionless values of the studied factors (using the recorded values of temperature, time, and pH) to avoid differences in the magnitude of the factor units (Table 1). Backward stepwise linear regression was applied to generate a second-order polynomial equation fitting the experimental ZnPP content containing only the significant terms (*p* < 0.05). The adjusted determination coefficient (R^2^adj) evaluated the model’s goodness-of-fit. The analysis of variance (ANOVA) was performed to investigate the statistical significance of the polynomial model using the significance *p*-value derived from the F-test at a 95% confidence level. Surface and contour plots derived from the polynomial model were used to determine the relationships between the variables. Additionally, the optimal conditions for ZnPP formation were determined by the desirability of the response between 0 and 1, aiming to maximize the ZnPP content while maintaining and, if possible, minimizing the microbial load. Desirability values close to 1 indicate more desirable conditions for ZnPP formation, and values close to 0 indicate more desirable conditions for total viable counts. The RSM-CCD was validated by comparing the predicted values obtained from the model with the observed values from the independent set of 14 additional experiments (see Section 2.4.2). 

## 3. Results and Discussion

### 3.1. Effect of the Addition of Disulfite and Organic Acids on ZnPP Formation and Microbial Growth

**Figure 1:**
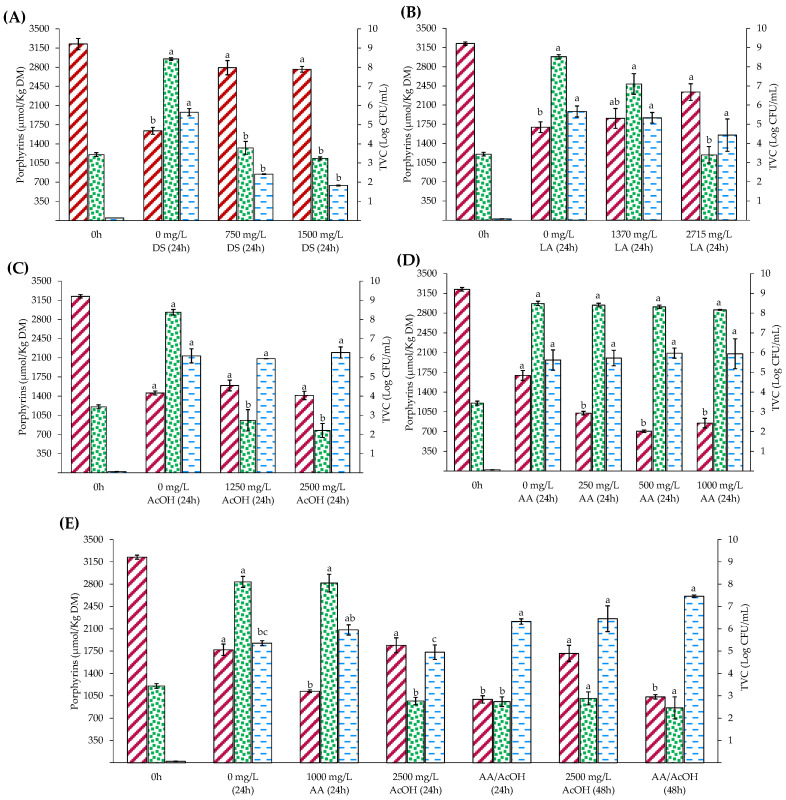
Effect of the addition of sodium disulfite (DS) (**A**), lactic acid (LA) (**B**), acetic acid (AcOH) (**C**), ascorbic acid (AA) (**D**), and combinations of ascorbic and acetic acids (AA/AcOH) (**E**) on heme content 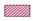, total viable counts of aerobic mesophilic bacteria (TVC) 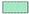, and Zn-protoporphyrin (ZnPP) content 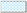. Liver homogenates (20%, *w*/*w*) were adjusted to pH 4.5 and incubated anaerobically at 37 °C in the dark for 24 h. The concentrations of ZnPP and heme are expressed on a dry-weight basis (% DM). TVC is expressed as log CFU/mL homogenate. Bars represent the standard error (n = 3). Significance differences among addition levels within the same incubation time are indicated by different letters (*p* < 0.05).

Figure 1 shows the porphyrin content (heme and ZnPP) and TVC in porcine liver homogenates before and after 24 h of anaerobic incubation at 37 °C. Heme was the dominant pigment before incubation, whereas the ZnPP content was negligible. After 24 h of incubation, the ZnPP content increased regardless of the added type and level of preservative. In all cases, the initial TVC was similar (ca. 3.5 logs CFU/mL) and, in the case of the controls, increased by 4–5 log units after 24 h of incubation. However, the preservative and its addition level led to different TVCs after 24 h of incubation. 

Regarding the effect of DS (Figure 1A), it was observed that heme loss was higher in the control than in the presence of DS after incubation. Heme loss is consistent with ZnPP formation. Accordingly, the highest ZnPP content was found in the control, ca. 1978 µmol ZnPP/kg DM, whereas the increase in DS led to a decrease in ZnPP content up to 641 µmol/kg DM at the highest level of addition. The DS-reducing capacity may prevent heme proteins such as hemoglobin from oxidizing, thus making it more challenging to release ferric heme from the apoprotein, a step described in the mechanism of ZnPP formation in ham [11]. It is possible that DS may decrease the activity of FECH, as this enzyme contains a redox-sensitive 2Fe-2S cluster and is dependent on NADH-cytochrome b5 reductase [26,27]. The antimicrobial effect of DS explains the maintenance of TVC at the initial levels, whereas, in the control group, the counts increased by five log units after incubation. However, the inhibition of ZnPP formation limits the use of DS to obtain a coloring ingredient based on this pigment.

After 24 h, the heme content decreased regardless of the addition of LA, whereas the ZnPP content increased in all cases (Figure 1B). There were no significant differences in ZnPP content, regardless of the LA addition level. However, there was a trend towards lower ZnPP content with higher levels of LA (*p* = 0.319). In addition, at the end of storage, the heme content at the highest level of LA addition was significantly higher than that in the control. In all cases, the sum of ZnPP and heme at the end of the process was higher than the initial values, which agrees with previous findings that PPIX may be formed independently, leading to the formation of ZnPP [16,28]. Compared to the control, an intermediate level of LA addition did not significantly prevent the increase in TVC, whereas the highest level of LA addition maintained the initial counts. The inhibition of microbial growth at the highest LA addition level could explain the higher heme content. Some Gram-negative and Gram-positive bacteria can take up heme through various mechanisms, including heme and heme protein receptors and the secretion of heme sequesters [29]. Heme can be used in different functions inside the cell, stored, or rapidly catabolized by heme oxygenase as an iron source [30]. Thus, it is possible that, depending on the conditions, different metabolic and catabolic processes coincide during the incubation of pork liver homogenates and determine the fate and final balance of the different porphyrins.

As for LA, adding AcOH (Figure 1C) resulted in a decreased heme content after incubation. Moreover, the formation of ZnPP was unaffected by the AcOH addition level, with an average value of approximately 2100 µmol/kg DM. This organic acid could prevent increased TVC irrespective of the concentration added. Wakamatsu et al. [31] studied the formation of ZnPP in infraspinatus porcine muscle homogenates with the pH adjusted from 4 to 6 by adding different organic acids and in the presence and absence of antibiotics. These authors reported an optimal pH of 4.75, whereas, in the absence of antibiotics, an additional optimal pH was observed at pH 5.5. Regardless of the addition of antibiotics, the pH adjustment at 4.75 with HCl, AcOH, and LA resulted in similar ZnPP contents after incubation, which agrees with our findings. However, in the presence of antibiotics, certain acids, such as malic acid, tartaric acid, and gluconic acid, led to higher ZnPP contents at pH 4.75 when compared with the same homogenate but adjusted with HCl. Accordingly, these authors concluded that the type of acid affected the growth of microorganisms in the pork homogenate after incubation, resulting in differences in the amount of ZnPP formed. The authors also reported that various bacterial strains present in meat homogenates could form ZnPP; consequently, the used of AcOH to adjust the pH may suppress the ZnPP-forming ability of microorganisms or their growth. The more substantial bacteriostatic effects of AcOH compared to LA can be explained not only by the level of addition, but also by the fact that organic acids are more effective as preservatives in a undissociated state and, under the pH conditions of the present study, the proportion of undissociated AcOH is higher than that of LA because of its higher pKa.

Taketani et al. [27] reported that the removal of ferrous ions from the porphyrin ring by FECH is NADH-dependent and occurs in combination with cytochrome b5 reductase, which is responsible for the ferric-heme reduction. The same authors also reported that adding reductants such as ascorbate facilitates the removal of iron ions from the heme moiety in the absence of NADH. The formation of PPIX may also explain the observed increase in the formation of ZnPP upon the addition of ascorbate [13,32]. We speculated that adding AA could benefit ZnPP formation by chelating and reducing various metal ions, such as Cu^2+^ and Fe^3+^, which have been reported to significantly inhibit FECH [33]. However, under our conditions, adding AA resulted in similar ZnPP content (ca. 2000 µmol/kg DM) compared with the control after 24 h of incubation (Figure 1D). Like the previously described preservatives, heme content decreased after 24 h of incubation, and the sum of heme and ZnPP was higher than that at the beginning of the incubation. The addition of AA did not affect TVC, which agrees with other studies that reported the inhibitory effects of specific vegetative pathogens at high levels of AA addition only [34,35]. Compared with the control, heme loss was higher in the presence of AA, whereas TVC was similar in both treatments. Therefore, increased heme loss may be attributed to the activity of heme oxygenase, which is largely present in the liver. This enzyme depends on NADPH-cytochrome P450 reductase; however, the latter can also be replaced with ascorbate to decompose heme [36,37].

Given that AcOH was shown to control microbial growth, we examined its potential synergistic effect with AA, as this was shown to maintain an ability to form ZnPP regardless of the increased heme loss during incubation. A decrease in heme content may be important to the development of meat products based on ZnPP; these meats may benefit from the lower heme content, given that heme is a well-known prooxidant compound. Figure 1E shows the effect of the combined addition on the ZnPP-forming capacity and TVC after 24 and 48 h of incubation. Adding AA alone or combined with AcOH resulted in higher heme loss than the control. However, the combined addition of AcOH and AA resulted in ZnPP content similar to that of AA alone. Moreover, a positive effect was observed compared with the control after 24 h of incubation, and the combined addition of AA and AcOH maintained TVC at the initial levels. After 48 h of incubation, ZnPP content continued to increase in the presence of AA and AcOH, whereas heme content remained unchanged, which supports alternative pathways for forming ZnPP. Therefore, a combination of AcOH and AA was selected to optimize the process using RSM to obtain a potential coloring ingredient.

The overall increase in the total porphyrin content after incubation contrasts previous findings in muscle meat products. For instance, in dry-cured ham produced without nitrifying agents, the sum of ZnPP and heme was 15% and 33% lower at the end of processing in the *biceps femoris* and *semimembranosus* muscles, respectively [6]. Similar results have been reported for nitrite-free dry fermented sausages [38]. These latter authors reported that total heme loss occurs gradually during formulation, and this process seems independent of pH, whereas the formation of ZnPP was proven to be influenced by pH. In an in vitro study, Akter et al. [39] reported that pork meat proteins with molecular weights higher than that of myoglobin in the soluble 10–30 kDa fraction contribute to ZnPP formation. Therefore, the origin and mechanisms of ZnPP in porcine meat and the liver and the effects of different organic compounds remain unclear. The role of microorganisms in ZnPP formation should not be disregarded, as several authors have reported their potential ability to produce ZnPP in meat products without adding nitrites or nitrates [31,40]. In meat extracts, *Pseudomonas fluorescens* can form ZnPP under aerobic conditions [41]. The bacteria in porcine livers are diverse and mainly consist of *Micrococcus*, *Streptococcus*, *Pediococcus*, *Staphylococcus*, and *Moraxella-Acintobacter* [42]. Therefore, further investigations are necessary to determine whether endogenous bacteria in the porcine liver can contribute to ZnPP formation or heme loss under the conditions examined here. However, upon adding organic acids, the formation of ZnPP seems to be relatively unaffected, whereas substantial changes occur in the TVC depending on the preservative and the level of addition. Thus, under the selected conditions involving the simultaneous addition of AA and AcOH, the formation of ZnPP seems to be independent of microbial growth.

### 3.2. Optimization of ZnPP Formation Using RSM

Table 2 shows the heme content, ZnPP content, and TVC for all 17 temperature, pH, and time combinations during ZnPP formation in the porcine liver homogenates. Interestingly, there was an overall increase in heme content with longer storage times (Table 2 and Supplementary Materials Figure S1). The release of insoluble heme, its interaction with other compounds, and its low recovery rate should be further investigated. However, these findings suggest that the rapid transformation into ZnPP could partly explain the lower heme content at the initial incubation stages (Supplementary Material Figure S1). It is also speculated that released iron may bind to iron regulatory proteins that induce heme synthesis [43,44]. This de novo heme synthesis may also explain the higher content of porphyrins (ZnPP and heme) after 24 h of incubation (Figure 1). Further studies are needed to confirm these mechanisms’ existence and clarify the origin of ZnPP under our conditions and in other meat systems.

To quantitatively describe the effect of the examined factors (incubation temperature, pH, and incubation time) on ZnPP formation, a second-order polynomial model was fitted to the ZnPP content data using a least square multivariate regression analysis (Table 3). Based on the ANOVA, the polynomial model obtained had a high adjusted R^2^, indicating that the model could explain 85.60% of the factors leading to the formation of ZnPP in porcine liver homogenates. According to the F-value (50.06) and associated low probability (*p* < 0.001), the model was highly significant. The goodness-of-fit of the ZnPP content regression model was also proven by plotting the observed data against the predicted response (Supplementary Material Figure S2).

**Figure 2:**
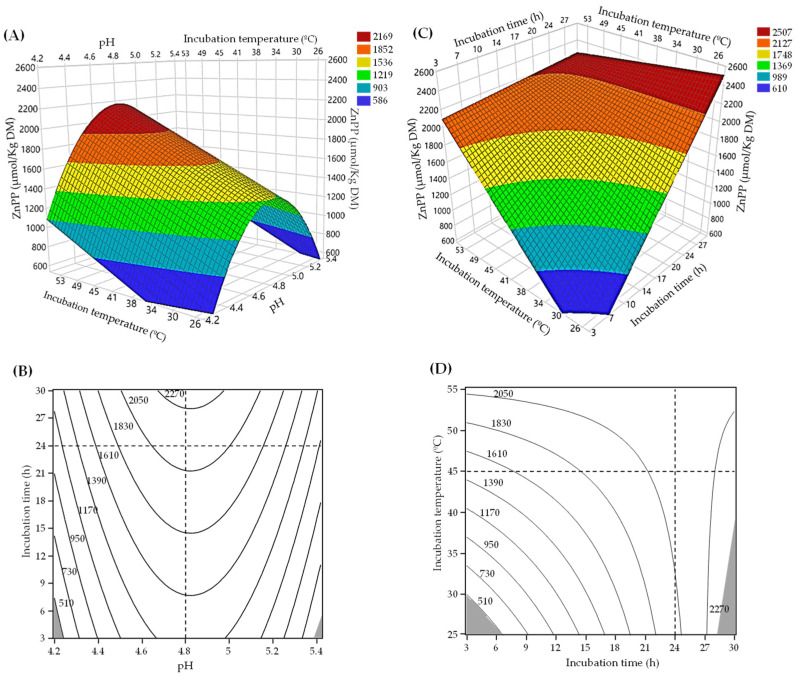
Formation of Zn-protoporphyrin (ZnPP) in porcine liver homogenates according to the developed model (see Table 3). The effect of the incubation temperature and pH on the formation of ZnPP is depicted as a response surface plot (**A**) and a contour plot (**B**). The effect of pH as a function of the incubation time and incubation temperature on ZnPP is depicted as a response surface plot (**C**) and a contour plot (**D**). In each plot, the factor not included is maintained at the central value of the central composite design, i.e., incubation time at 16.5 h (**A**) and pH at 4.8 (**B**). The dotted lines indicate the optimal conditions for forming ZnPP from porcine liver homogenates (at pH 4.8 and 45 °C for 24 h under anaerobiosis). The grey area in the counter plots (**B**,**D**) indicates the limit for the ZnPP values according to the model (see Table 3).

The surface and contour plots of ZnPP formation are shown in Figure 2. The response surfaces describe how all three factors affect the formation of ZnPP. ZnPP formation was dependent on the incubation temperature and pH (Figure 2A). The role of pH was independent of incubation temperature, as no significant coefficient represented the interaction between these two parameters (Table 3). Thus, the quadratic pH term markedly contributed to ZnPP formation, reaching its maximum at pH 4.8. This optimal pH agrees with the reported pH range (4.5–5.5) in different porcine muscles and the liver [16,17]. It can also be observed that the formation of ZnPP increases with temperature, with a maximum between 45 and 55 °C. This finding agrees with the reported increase in the formation of ZnPP when incubated at physiological or slightly higher temperatures [17,33]. Therefore, the incubation temperature plays a vital role in the formation of ZnPP, but to a lesser extent than the pH, as indicated by the lower beta value (Table 3). In addition, the contour plot in Figure 2B indicates that maintaining the pH at 4.8 makes it feasible to obtain ZnPP contents of over 2050 µmol/kg DM over a broad range of temperatures (30–55 °C).

The effects of the incubation temperature and time on the formation of ZnPP are depicted in Figure 2C,D. In the range tested and at pH 4.8, ZnPP formation increased at higher temperatures and incubation times. Thus, it is possible to achieve high ZnPP content with both incubations at high temperatures for shorter times and at low temperatures for longer incubation times. However, when the standardized regression coefficients were compared, the incubation time term (beta = −0.602) was higher than the incubation temperature term (beta = −0.3368). In agreement with these findings, several studies have reported the crucial effects of pH, temperature, and incubation time on the ZnPP formation capacity [17,28,33].

Table 2 shows that, in general, TVC remained low, with small changes after different incubation times (ranging between 2.85 and 3.54 log CFU/mL), which agrees with the observed antimicrobial effect of AcOH (Section 3.1). However, there were two exceptions: runs 6 and 8, where the microbial load was higher (6.18 and 5.26 log CFU/mL, respectively). AcOH’s lack of an antibacterial effect in runs 6 and 8 can be attributed to these runs’ higher pH values (5.4 and 5.2, respectively) and optimal growth temperatures (32 and 37 °C, respectively). Several physical, physicochemical, and microbiological factors play a crucial role in determining the microbial stability of food products, which should be considered when designing a process based on hurdle technology to obtain safe food products [45]. At the optimal pH of 4.8, temperatures above 40 °C were sufficient to maintain microbial counts below 3 logs CFU/mL, regardless of the incubation time (Supplementary Materials Figure S3). Therefore, maximizing the formation of ZnPP while controlling microbial growth in a range of incubation times and temperatures seems feasible.

#### 3.2.1. Selection and Validation of Optimal Conditions for ZnPP Formation

The obtained model (Table 3) maximized ZnPP formation while constraining the total viable counts to ≤3 logs CFU/mL. The desired optimal conditions (t, T, pH = −1.000, −1.000, 1.84 × 10^−10^) corresponded to 24.5 h of incubation at 49 °C and a pH of 4.80. Under these selected conditions, the ZnPP model predicted a ZnPP content of 2174 µmol/kg DM and a TVC of 2.32 log CFU/mL. These conditions were deemed sufficient for developing a potential ingredient because the obtained ZnPP content was 24-fold higher than that found in nitrite-free Parma dry-cured hams [46]. The predicted TVC remained at the initial counts, similar to those found in other raw meat products. For practical reasons and considering that the model has minimal differences in terms of TVC and ZnPP content, we ultimately decided to select anaerobic incubation at pH 4.8 and 45 °C for 24 h. Under the selected optimal conditions, the bacterial relative abundance is mainly composed of lactic acid bacteria species, with the most abundant being *Lactobacillus johnsonii* (62.64%), *Limosilactobacillus reuteri* (34.89%), *Limosilactobacillus mucosae* (6.34%), and *Lactobacillus amylovorus* (5.43%) [47].

Fourteen experiments conducted to validate the selected optimal conditions for ZnPP formation were plotted against the predicted values obtained using the ZnPP model for validating the developed model’s adequacy (Supplementary Materials Figure S2). The predicted and observed values were compared, and the residual and percentage errors were calculated. The latter ranges between 18 and 12%. These results indicate that the developed model tends to overestimate the ZnPP content. It is worth noting that several intrinsic and extrinsic factors may determine the formation of ZnPP in liver and muscle foods; these factors still need to be fully elucidated. Thus, FECH may play a crucial role in the potential ability to form ZnPP during incubation. Under the selected optimal conditions, the activity of FECH (measured as Zn-chelatase) is at its maximum at the initial time (87 ± 4.3 nmol/g liver DM·min). This activity in fresh homogenates is consistent with that reported by De Maere et al. [15]. However, this activity progressively decreases with the course of the incubation (36 ± 1.2 and 3 ± 0.4 nmol/g liver DM·min after 15 and 30 h of incubation, respectively), which seems coincident with the rapid ZnPP formation and, after 15–18 h, progressive stabilization when incubated at 45 °C (Figure 2D). Additionally, in the validation process, the TVC was found to be lower than 3 logs CFU/mL, in compliance with the requirements of the model.

#### 3.2.2. Definition and Safety Aspects of the Potential Coloring Ingredient

The formation of ZnPP from porcine liver homogenates resulted in a directly functional aqueous ingredient containing 3% protein and 1717 ± 53 µmol/kg DM. In aqueous meat extracts, ZnPP appears to be mainly formed after the direct substitution of Fe by Zn ions within myoglobin; however, during incubation, the porphyrin moiety can be released, leading to the appearance of unbound ZnPP [48,49]. The mechanism proposed for the manufacturing of dry-cured ham implies the release of the heme moiety from hemoglobin; this then transforms into unbound ZnPP, which can be non-enzymatically inserted into the intact apohemoglobin [11]. Under our conditions, regardless of the mechanism, the presence of free ZnPP explains the observation that the ZnPP formed remained in the insoluble protein fraction after centrifugation. However, this insoluble fraction containing ZnPP was considered suitable for the maximum coloring capacity and protein recovery in the development of meat products without the addition of nitrifying agents. Accordingly, in comparison to the liver homogenate obtained under the selected conditions after 24 h of incubation, its centrifugation resulted in a 4-fold richer ZnPP-based potential coloring ingredient (ca. 6800 µmol/kg DM), with 12% protein, 6% fat, and 81% moisture.

A challenge test was performed to study the behavior of relevant pathogens present in the raw materials (porcine liver) during the ZnPP formation process to assess the microbiological safety of the resulting ingredient. Immediately after inoculation, counts were 7.5 ± 0.10 log CFU/mL and 7.86 ± 0.08 log CFU/mL for *L. monocytogenes* and *S. enterica*, respectively. At the end of the process and after centrifugation to obtain the ZnPP-based ingredient, none of the vegetative pathogens were detected, indicating that the conditions applied were harsh enough to inhibit growth and even caused significant inactivation (>6 log reduction) for both *L. monocytogenes* and *S. enterica*. Although the temperature used in the ZnPP process formation was at or close to the maximum boundary allowing growth for *L. monocytogenes* (Tmax = 45 °C) and *Salmonella* (Tmax = 46.2 °C) when all the other factors were optimal [50], the concentration of AcOH was probably the most relevant factor for the inhibition of growth. The pH of 4.8 used for ZnPP formation equals the pKa of the AcOH. Thus, 50% of the added AcOH was in its active antimicrobial form, i.e., 1250 mg/L. This concentration is above the minimal inhibitory concentration (MIC) of AcOH reported for *L. monocytogenes* (MIC = 10.3 mM, Mejlholm and Dalgaard [51]) and *Salmonella* (MIC = 17.05 mM, Beier et al. [52]). Moreover, it is well known that, when vegetative bacteria are exposed to physicochemical conditions that do not allow for growth, they die more rapidly when the temperature limits of growth and/(or) multiple hurdles are combined. At these temperatures, microorganisms attempt to overcome and repair the damage caused by hostile conditions, and the increasing energy demands to maintain internal homeostasis under stress conditions cause cellular death (inactivation) due to metabolic exhaustion mechanisms [53].

Spores of *C. perfringens* could not germinate and grow during the production process of the ZnPP-based ingredient despite the anaerobic conditions and the temperature, which was close to the optimal temperature for this growth pathogen (Topt = 43–47 °C, ICMSF [50]). However, none of the production process steps included a heat treatment to activate spore germination, and the pH of the medium below the minimum growth pH of *C. perfringens* (5.5–5.8, ICMSF [50]) would, in any case, inhibit its growth. However, in contrast to vegetative pathogens, *C. perfringens* spores were resistant to the ingredient retention process, remaining at the initial inoculated levels (6.50 log CFU/mL). Nevertheless, the survival of spores that are potentially present in liver homogenates and which produce ZnPP-based ingredients should not be an unassailable drawback for developing nitrite-free meat products. It may require specific processing technologies (e.g., thermal treatments) and/or the definition of an appropriate hurdle technology to prevent the growth of *C. perfringens* in developing nitrite-free meat products using ZnPP-based ingredients.

## 4. Conclusions

The addition of AcOH alone or in combination with AA allowed for the formation of ZnPP after incubation while maintaining TVC. The optimal conditions for maximum ZnPP formation and microbial growth control involve combining these organic acids at pH 4.8 and incubating at 45 °C for 24 h under anaerobiosis. Under these conditions, a considerable amount of ZnPP was formed without the risk of microbial growth but while considerably decreasing the levels of the studied vegetative foodborne pathogens. After processing, liver homogenates could be appropriate for further use as potential coloring ingredients for the development of cured-meat-like products without adding nitrites or nitrates. Further research is needed to examine the coloring capacity of the developed ingredients and their possible applications in the food industry, thus contributing to the valorization of porcine livers.

## Figures and Tables

**Table 1:** Factors and levels of the central composite design (CCD) for the optimization of the response surface methodology.

Factors			Levels		
	−1.68	−1	0	+1	+1.68
Temperature (°C)	25	31	40	49	55
pH	4.2	4.4	4.8	5.2	5.4
Time (h)	3	8.5	16.5	24.5	30

**Table 2:** Central composite design (CCD) results were obtained in each trial for the porcine liver homogenates according to different incubation temperatures, pH levels, and incubation time conditions.

Run	Temperature (°C) ^1^	pH ^1^	Time (h) ^1^	Heme (µmol/kg) ^2^	ZnPP (µmol/kg) ^2^	TVC (Log CFU/mL) ^2^
1	55 (50)	4.80 (4.79)	16.5 (17.0)	515 ± 7	2195 ± 20	2.85 ± 0.01
2	40 (37)	4.20 (4.23)	16.5 (16.6)	310 ± 14	643 ± 1	3.14 ± 0.14
3	40 (37)	4.80 (4.79)	16.5 (16.7)	318 ± 12	1754 ± 51	3.23 ± 0.05
4	40 (37)	4.80 (4.79)	3.0 (3.2)	333 ± 51	617 ± 9	2.97 ± 0.02
5	40 (37)	4.80 (4.79)	16.5 (16.7)	358 ± 13	1829 ± 13	3.13 ± 0.05
6	40 (37)	5.40 (5.41)	16.5 (16.7)	382 ± 17	788 ± 4	6.18 ± 0.21
7	31 (32)	5.20 (5.21)	8.5 (8.7)	357 ± 16	1006 ± 15	2.95 ± 0.05
8	31 (32)	5.20 (5.21)	24.5 (25.0)	1422 ± 21	1380 ± 11	5.26 ± 0.14
9	40 (37)	4.80 (4.79)	30.0 (30.0)	940 ± 22	2538 ± 73	3.39 ± 0.25
10	49 (47)	5.20 (5.21)	8.5 (8.3)	335 ± 86	1185 ± 52	2.87 ± 0.09
11	49 (47)	5.20 (5.21)	24.5 (25.0)	1258 ± 30	1699 ± 45	2.95 ± 0.01
12	49 (47)	4.40 (4.40)	8.5 (8.3)	381 ± 21	1347 ± 42	3.02 ± 0.32
13	40 (37)	4.80 (4.79)	16.5 (16.7)	499 ± 153	1728 ± 22	3.22 ± 0.04
14	31 (32)	4.40 (4.40)	24.5 (25.0)	1067 ± 17	1798 ± 25	3.16 ± 0.01
15	25 (25)	4.80 (4.79)	16.5 (17.0)	617 ± 143	1200 ±16	3.54 ± 0.01
16	49 (47)	4.40 (4.40)	24.5 (25.0)	1157 ± 1	1468 ± 1	2.77 ± 0.07
17	31 (32)	4.40 (4.40)	8.5 (8.7)	330 ± 5	513 ± 25	3.20 ± 0.12

^1^ The columns for the independent factors, that is, temperature, pH, and incubation time, report the target values according to the central composite design (Table 1); the actual recorded values for each run are reported in brackets. ^2^ response variables are expressed as the mean ± SE (n = 2) in dry-weight basis.

**Table 3:** Summary of the analysis of variance (ANOVA) of the second-order polynomial model for ZnPP formation in porcine liver homogenates.

Term	Degrees of Freedom	Sum of Squares	F	Coefficient	Standard Error	t Value	*p*-Value	Standard Beta
Constant				1755.930	53.9589	32.543	<0.0001	0.000
Incubation time (t)	1	3585.966	78.97	−366.049	41.1925	−8.886	<0.0001	−0.602
Incubation temperature (T°C)	1	1166.106	25.68	−242.202	47.795	−5.067	<0.0001	−0.336
t · T°C	1	463.142	10.20	−189.832	59.441	−3.194	0.0034	−0.217
pH^2^	1	3357.405	73.94	−350.978	40.818	−8.599	<0.0001	−0.569
Model	4	9083.892	50.06				<0.0001	
Pure Error	12	32.931						
Total	29	1325.367						

R^2^ = 0.8735; R^2^ adj. = 0.8560; RSME = 213.096.

## Data Availability

The data presented in this study are available on request from the corresponding author.

## Supplementary Materials

The following supporting information can be downloaded at: https://www.mdpi.com/article/10.3390/foods12091903/s1, Figure S1: Heme content in porcine liver homogenates according to the Zn-protoporphyrin formation model (Table 3). The effects of incubation time and temperature on heme content are depicted as a response surface plot (A). The effects of incubation time and pH on heme content are depicted as a response surface plot (B). The effects of incubation temperature and pH on heme content are depicted as a response surface plot (C). In each surface plot, the factor not included is kept at the central value of the central composite design, that is, pH at 4.8 (A), incubation temperature at 40 °C (B), and incubation time at 16.5 h (A). Figure S2: Observed ZnPP content versus predicted values using the regression model (see Table 3). The selected ZnPP optimal formation conditions (45 °C, 24 h, and pH 4.8 under anaerobiosis) were validated using the same conditions and pool of liver samples as described in the response surface methodology, and eleven independent pools of porcine livers (n = 4–7) were used in the pilot scale-up (4–8 L) of the process. Figure S3: Total viable counts of aerobic mesophilic bacteria (TVC) in porcine liver homogenates according to the model for Zn-protoporphyrin formation (see Table 3). The effects of incubation temperature and pH on TVC growth are depicted as a response surface plot (A). The effects of the incubation time and temperature on TVC growth are depicted as a response surface plot (B). The effects of incubation time and pH on TVC growth are depicted as a response surface plot (C). In each surface plot, the factor not included was kept at the central value of the central composite design, that is, incubation time at 16.5 h (A), pH at 4.8 (B), and incubation temperature at 40 °C (C).
