# Supplementary material for: Utilization of Porcine Livers through the Formation of Zn-Protoporphyrin Pigment Optimized by a Response Surface Methodology
Source: Foods. 2023 May 6;12(9):1903. doi: 10.3390/foods12091903 (PMC10178239; doi:10.3390/foods12091903)
Supplement: Supplementary file 1 [file foods-12-01903-s001.zip › foods-2373399-supplementary.pdf]

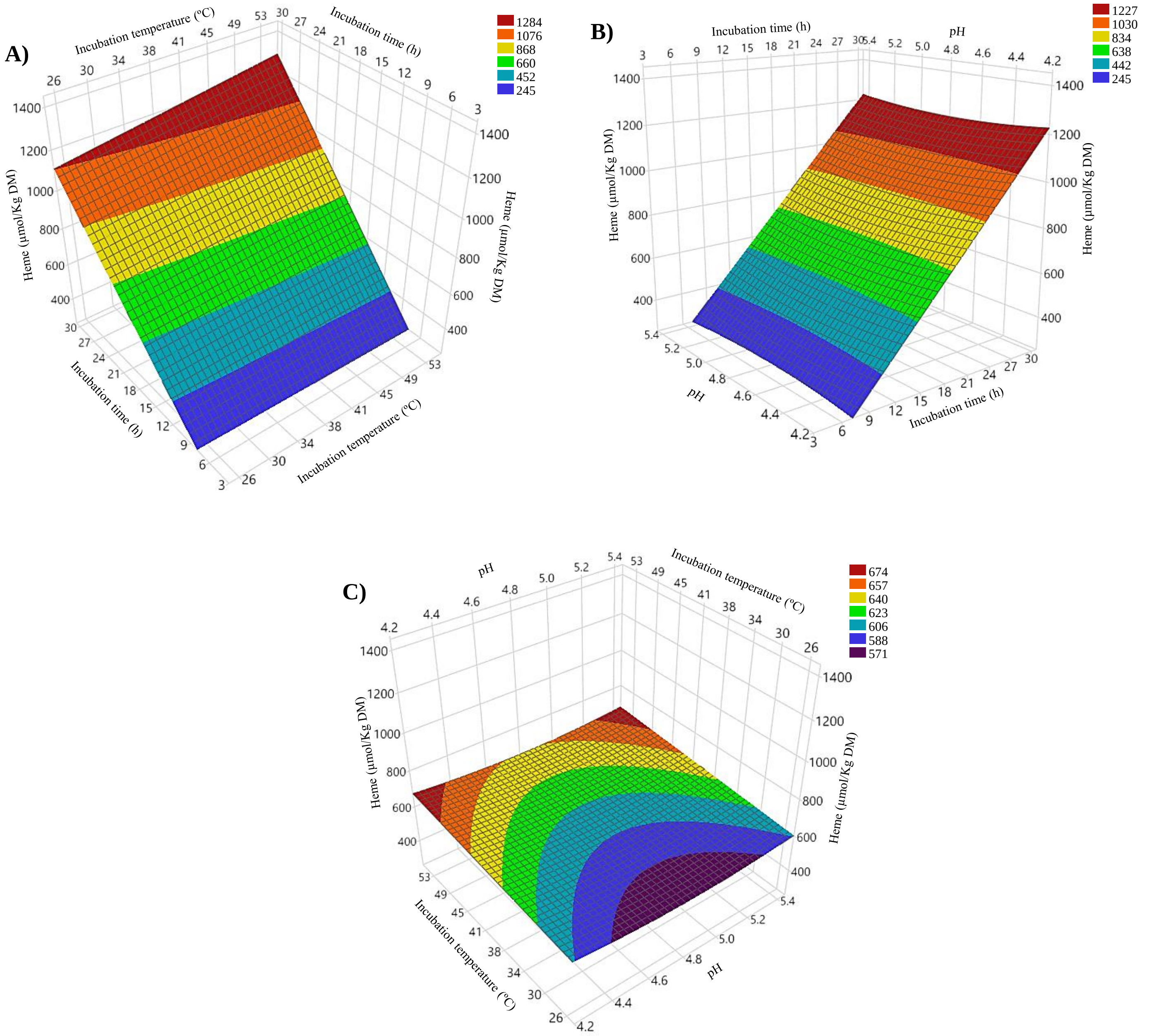

Figure S1. Heme content in porcine liver homogenates according to the Zn-protoporphyrin formation mod-el (Table 3). The effects of incubation time and temperature on heme content are depicted as a re-sponse surface plot (A). The effects of incubation time and pH on heme content are depicted as a response surface plot (B). The effects of incubation temperature and pH on heme content are de-picted as a response surface plot (C). In each surface plot, the factor not included is kept at the central value of the central composite design, that is, pH at 4.8 (A), incubation temperature at 40 °C (B), and incubation time at 16.5 h (A).

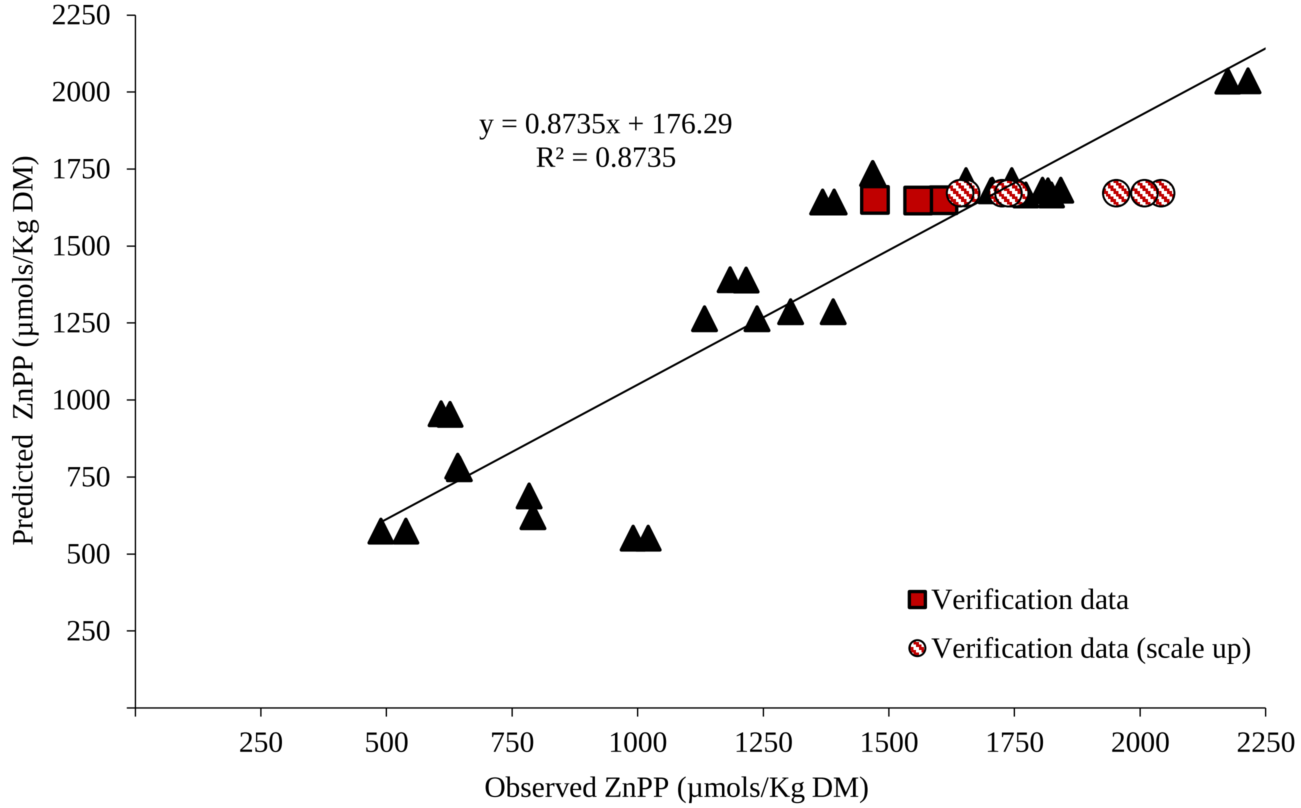

Figure S2. Observed ZnPP content versus predicted values using the regression model (see Table 3). The selected ZnPP optimal formation conditions (45 °C, 24 h, and pH 4.8 under anaerobiosis) were vali-dated using the same conditions and pool of liver samples as described in the response surface methodology, and eleven independent pools of porcine livers (n = 4–7) were used in the pilot scale-up (4–8 L) of the process.

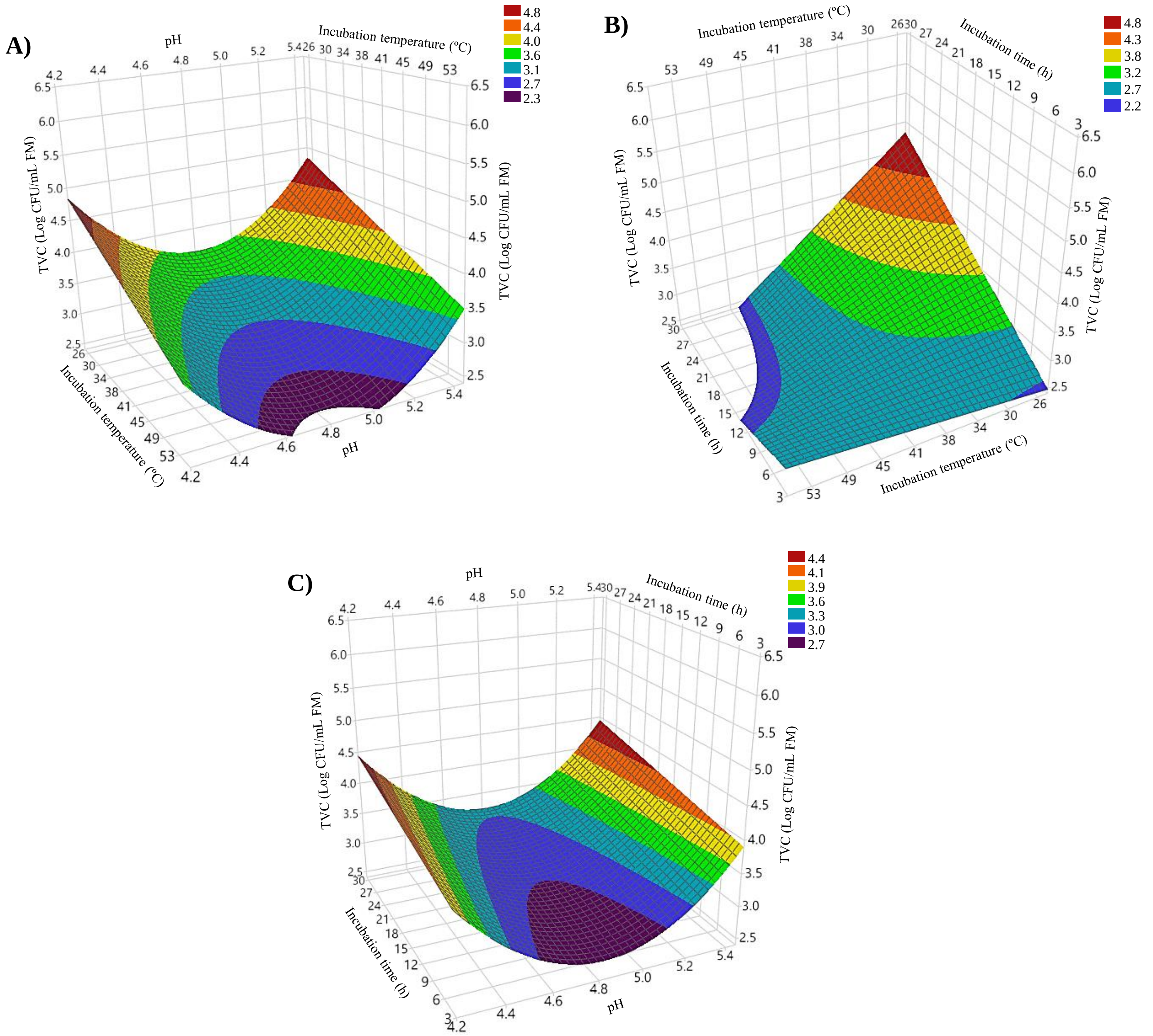

Figure S3. Total viable counts of aerobic mesophilic bacteria (TVC) in porcine liver homogenates according to the model for Zn-protoporphyrin formation (see Table 3). The effects of incubation tempera-ture and pH on TVC growth are depicted as a response surface plot (A). The effects of the incuba-tion time and temperature on TVC growth are depicted as a response surface plot (B). The effects of incubation time and pH on TVC growth are depicted as a response surface plot (C). In each surface plot, the factor not included was kept at the central value of the central composite design, that is, incubation time at 16.5 h (A), pH at 4.8 (B), and incuba-tion temperature at 40 °C (C).
